# Isolation and Characterization of *Salmonella* Jumbo-Phage pSal-SNUABM-04

**DOI:** 10.3390/v13010027

**Published:** 2020-12-25

**Authors:** Jun Kwon, Sang Guen Kim, Hyoun Joong Kim, Sib Sankar Giri, Sang Wha Kim, Sung Bin Lee, Se Chang Park

**Affiliations:** Laboratory of Aquatic Biomedicine, College of Veterinary Medicine and Research Institute for Veterinary Science, Seoul National University, Seoul 08826, Korea; kjun1002@snu.ac.kr (J.K.); imagine5180@gmail.com (S.G.K.); hjoong1@nate.com (H.J.K.); giribiotek@gmail.com (S.S.G.); kasey.kim90@gmail.com (S.W.K.); lsbin1129@naver.com (S.B.L.)

**Keywords:** bacteriophage, jumbo-phage, reptile-associated salmonellosis, phage therapy, bacteriophage genome

## Abstract

The increasing emergence of antimicrobial resistance has become a global issue. Therefore, many researchers have attempted to develop alternative antibiotics. One promising alternative is bacteriophage. In this study, we focused on a jumbo-phage infecting *Salmonella* isolated from exotic pet markets. Using a *Salmonella* strain isolated from reptiles as a host, we isolated and characterized the novel jumbo-bacteriophage pSal-SNUABM-04. This phage was investigated in terms of its morphology, host infectivity, growth and lysis kinetics, and genome. The phage was classified as *Myoviridae* based on its morphological traits and showed a comparatively wide host range. The lysis efficacy test showed that the phage can inhibit bacterial growth in the planktonic state. Genetic analysis revealed that the phage possesses a 239,626-base pair genome with 280 putative open reading frames, 76 of which have a predicted function and 195 of which have none. By genome comparison with other jumbo phages, the phage was designated as a novel member of *Machinavirus* composed of *Erwnina* phages.

## 1. Introduction

Bacteriophages are virus that can infect and lyse bacterial cells [1,2]. These bacterial viruses are considered promising alternatives to antibiotics because of their unique characteristics [1,2]. Phages have often a narrow range of host specificity [1,2], enabling their application to eradicate target bacteria without disrupting the normal flora [1,2]. Furthermore, bacteriophages show remarkable biosafety even with long-term administration [1,2]. These features make bacteriophages among the best alternatives to antibiotics [2].

Phages with genomes larger than 200 kbp are known as jumbo-phages [3]. Because of their large genomes, jumbo-phages are highly diverse [3,4] and possess a larger number of genes in comparison to phages with shorter genomes [3]. These gene products may reduce the utilization of host proteins and make them less dependent on the host strains, making giant phages enable to have a wider host range [3,5]. However, even until recently many jumbo-phage gene products remained as hypothetical proteins. Although many jumbo-phage genomes remain uncharacterized, these bacterial viruses provide important clues for understanding bacteriophages in general [3,4,5].

In this study, we isolated and characterized a novel jumbo-bacteriophage, pSal-SNUABM-04, which is virulent to *Salmonella* sp. We examined the biological and genomic features of this jumbo-phage and compared its genomic features to those of other jumbo-phages.

## 2. Materials and Methods 

### 2.1. Bacterial Strains Used in This Study 

The *Salmonella* strain was isolated from pet reptiles. Samples were collected by swabbing the reptile skins and cloaca. Sixty skin swabs and 20 cloacal swabs were performed. After overnight enrichment in Rappaport-Vassiliadis R10 broth (Difco, Detroit, MI, USA) at 27 °C, the enrichment medium was spread on xylose lysine deoxycholate agar (Difco) and incubated overnight at 27 °C. Suspected colonies were sub-cultured by the streak plate method on fresh xylose lysine deoxycholate agar three times. The isolates were identified by 16S rRNA gene sequencing performed by Macrogen (Seoul, Korea). Fourteen of the suspected isolates were confirmed to be *Salmonella*. The bacteria were stored at −70 °C in tryptic soy broth (TSB; Difco) containing 15% glycerol until use.

### 2.2. Isolation and Characterization of the Phage pSal-SNUABM-04

The *S. enterica* strain Sal-SNUABM-svn1, isolated from a Savannah monitor *(Varanus exanthematicus*) cloacal swab sample, was used to isolate the bacteriophage as described by Kim [1]. Briefly, environmental water samples collected in South Korea were filtered through 0.45 µm membrane filters (Merck Millipore, Billerica, MA, USA). The filtered samples were mixed with TSB (1:1) into which an overnight cultured host strain (1%, *v/v*) was inoculated. The suspension was incubated for 24 h at 27 °C. The presence of bacteriophage was verified by the conventional double-layer agar technique. Double-layered agar plates were incubated overnight at 27 °C. After observing plaque formation, single plaques were isolated with a Pasteur pipette 5 times. The isolated bacteriophage was designated as pSal-SNUABM-04.

The phage was characterized as described by Kim [1]. For phage adsorption analysis, the host bacterial strain in the exponential phase was infected with 10 μL phage suspension (final multiplicity of infection, MOI, of 0.001), and the mixture was incubated at 27 °C. To titrate un-adsorbed phages, aliquots (100 μL) were collected at 0, 0.5, 1, 2, 3, 5, 7, 10, 15, 20, and 30 min after phage infection. The aliquots were centrifuged at 12,000× *g* for 3 min, and the supernatants were diluted in 900 μL phosphate-buffered saline and cultured by the conventional double-layered-agar method. A one-step growth curve was constructed, and the phage lysate was inoculated into the host bacterial strain culture in the exponential phase. The final MOI of the phage and bacterial cell mixture was 0.001. After more than 95% of the phages had adsorbed for 15 min according to the results of adsorption analysis, the mixtures were centrifuged at 12,000× *g* for 3 min. The supernatant was discarded, and the remaining bacterial pellet was resuspended in preheated TSB and incubated at 27 °C with shaking at 150 rpm. Aliquots (100 μL) were collected every 10 min for 140 min. Plaque-forming units were determined using the double-layered agar method. All experiments were performed in triplicate.

The host range was tested using the double-layered agar method. Bacterial strains isolated from reptiles in this study were used. The phage concentration used in this experiment was 1.0 × 10^6^ PFU/mL and the experiments were performed in triplicate.

### 2.3. Electron Microscopy of the Phage pSal-SNUABM-04 

Transmission electron microscopy (TEM) of pSal-SNUABM-04 was performed using a Talos L120C (FEI, Hillsboro, OR, USA). For TEM, phage concentration and purification were performed by polyethylene glycol 8000-NaCl precipitation in SM buffer and the phage was stained with 2% uranyl acetate. The dimensions of individual phages were measured.

### 2.4. In Vitro Planktonic Bacterial Lysis Assay

A planktonic bacterial cell lysis assay was conducted to evaluate the lytic efficacy against the host strain. Under MOIs of 0.1, 1, and 10, we analyzed the turbid and clear lysis patterns of the phage. Overnight-cultured bacteria were inoculated into TSB to prepare a bacterial suspension of 10^8^ colony-forming units/mL. Next, 200 μL of the bacterial suspension was dispensed into 96-well plates. The phage lysates were inoculated to reach the final MOIs mentioned above and the plates were cultured at 27 °C with shaking at 150 rpm. The turbid and clear patterns of phage–bacteria suspensions were determined by measuring the OD_600_ with a VersaMax Microplate Reader (Molecular Devices, Sunnyvale, CA, USA). The measurement intervals were 30 min until 18 h, and 60 min from 18 to 24 h. After the assays, the mixtures were sub-cultured 3 times, and the phage sensitivity of the cultured bacteria was tested by the double-layered agar method. The experiments were performed in triplicate.

### 2.5. Evaluation of Phage Stability under Different Thermal and pH Conditions

Phage stability was evaluated under different thermal and pH conditions. To test the pH stability of the phage, the phage lysate was inoculated in 1 mL of TSB prepared at different pH levels (pH 2.0, 4.0, 5.0, 7.0, and 9.0) adjusted with 1 M HCl and 1 M NaOH solutions. The mixtures were incubated at 27 °C for 2 h, and phage titration was performed by dilution and conventional double-layered plating. The thermal stress stability of the phage was evaluated at different temperatures (4 °C, 20 °C, 27 °C, and 37 °C). After incubating the phage suspensions at different temperatures for 2 h, titration was performed by dilution and plating. All analyses were performed with Sigmaplot 14.0 software (Systat Software Inc., IL, USA) using analysis of variance with Dunnett’s post-hoc test. *P* values < 0.05 were considered significantly different.

### 2.6. Phage Sequencing and Genome Analysis

Phage DNA extraction was performed as described by Kim [1]. Purified phage genomic DNA was sequenced on an Illumina Hiseq2500 platform (San Diego, CA, USA) at Genotech (Daejeon, Korea). We used Unipro UGENE v35.0 to trim and assemble the reads. For putative open reading frame (ORF) prediction, Rapid Annotation using Subsystem Technology v2.0 (RAST), GeneMarkS v4.28, and protein BLAST were used [6,7]. tRNA detection was performed using tRNAscan-SE v2.0 [8]. The functions of the predicted ORFs were verified by BLAST searching. The conserved domains of the phage genome were searched by HHpred tool [9].

### 2.7. Comparative Genome Analysis

For phylogenetic analysis, the genome sequences of the jumbo phages were obtained from the GenBank database and aligned using Clustal W [10]. For single genome phylogeny analysis, we used sequences of the major capsid protein and terminase large subunit genes. Phylogenetic trees were constructed in MEGA v10.1.8 software using the maximum likelihood method with 1000 bootstrap replications [11]. For the whole-genome phylogenetic tree, we used the Genome-BLAST Distance Phylogeny method in the Virus Classification and Tree Building Online Resource (VICTOR) [12]. The resulting intergenomic distances (including 100 replicates each) were used to infer a balanced minimum evolution tree with branch support via FASTME including subtree pruning and regrafting postprocessing for the formula D0 [13]. The tree was visualized using FigTree [14]. Dot plots were drawn using Gepard at a word size of 10 [15]. The EZBiocloud ANI calculator was used to calculate the average nucleotide identity [16]. Comparative genome analysis of Machinavirus and pSal-SNUABM-04 was performed using Mauve [17].

## 3. Results and discussions

### 3.1. Bacterial Strains Used in This Study

Fourteen strains of *Salmonella enterica* bacteria were isolated from the swab samples. Among them, Sal-SNUABM-svn1 was used as the host strain (Table 1). The phage showed a wide host range towards reptile Salmonella [71.42% (10/14)]. The ability of the phage to infect a wide host range likely involves many genes encoding factors important in DNA replication and nucleotide metabolism, similar to other jumbo-phages examined in previous studies [3,5].

### 3.2. Biological Features of the Phage pSal-SNUABM-04

**Figure 1:**
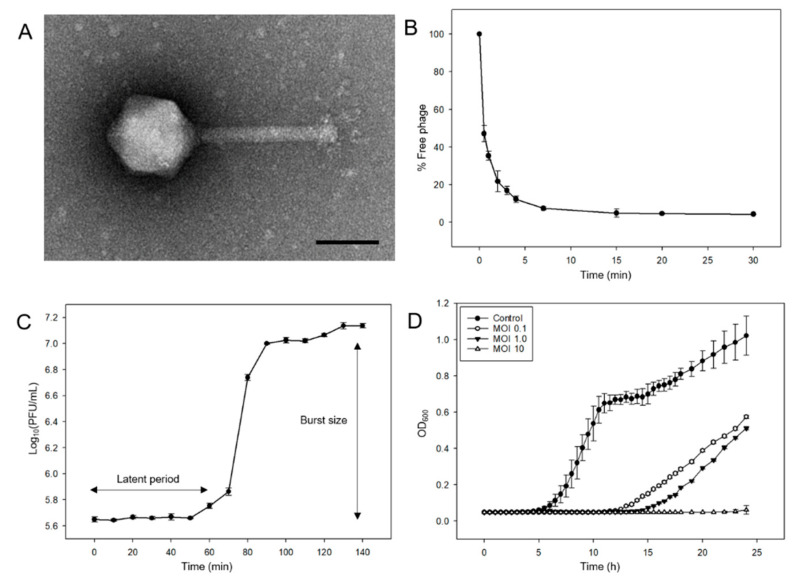
Morphological and biological features of phage pSal-SNUABM-04. (**A**) Transmission electron micrograph of pSal-SNUABM-04. Scale bar = 100 nm. (**B**) Adsorption assay of pSal-SNUABM-04 to host strain Sal-SNUABM-svn1. (**C**) One-step growth curve of pSal-SNUABM-04 to host bacterial strain. (**D**) In vitro planktonic cell lysis efficacy assay of pSal-SNUABM-04 at MOI of 0.1, 1, and 10 against host bacterial strain.

*Salmonella* phages were isolated from water samples collected from the Nam-river, South Korea. Phage morphology was determined by TEM and classified based on the criteria proposed by Ackermann [18]. Phage pSal-SNUABM-04 was designated in the *Myoviridae* family and had an icosahedral head with a diameter of 80 ± 3 nm and tail length of 116± 10 nm (Figure 1A).

The adsorption test showed that 95% of the phage was adsorbed in 15 min (Figure 1B). The phage adsorption constant k was calculated as described in a previous study [19], *k* = 3.76 × 10^−10^ mL/min. The latent period of phage pSal-SNUABM-04 was 60 min and the burst size was 29.11 (Figure 1C).

The results of the cell lysis assay against planktonic bacterial cells are shown in Figure 1D. In contrast to the continuous increase in the control group (non-phage-treated group), the phage-treated groups showed significant decreases in OD_600_ values. Gradual bacterial growth was observed in the 0.1 and 1 MOI groups. However, when the phage was treated at a high concentration (MOI = 10), bacterial growth was inhibited. All cultured bacteria from the cell lysis assays showed sensitivity to phage pSal-SNUABM-04. 

**Figure 2:**
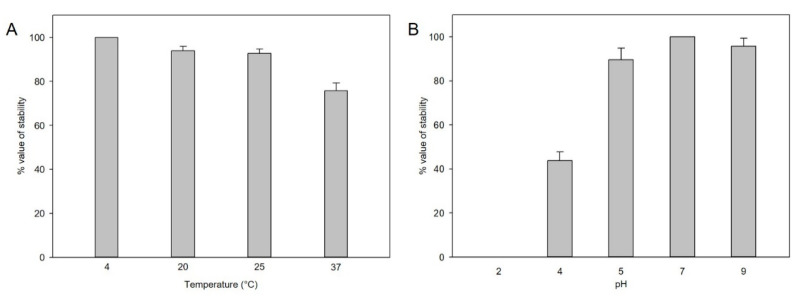
(**A**) Thermal stability of pSal-SNUABM-04. Phage lysates were incubated under different thermal conditions for 2 h. (**B**) pH stability of pSal-SNUABM-04. Phage lysates were incubated under different pH conditions for 2 h. Asterisks indicate statistically significant differences (*p* < 0.05), *n* = 3.

For plaque-forming unit determination, phage stability under different thermal and pH conditions was analyzed. At different pH levels, the phage titers decreased, particularly at lower pH levels of pH 4–5, but not significantly at pH 5–9 for 2 h (Figure 2A). In the thermal stability test, the phages were stable at 4 °C, 25 °C, and 27 °C, whereas stability was decreased at 37 °C within 2 h (Figure 2B).

### 3.3. General Features of the Phage pSal-SNUABM-04 Genome

**Figure 3:**
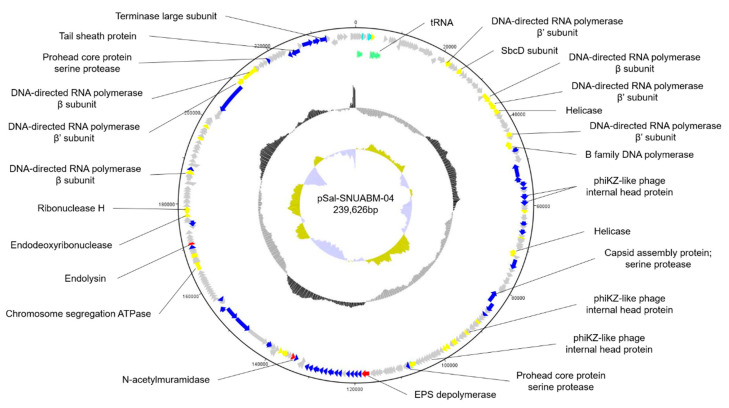
Genome map of pSal-SNUABM-04. The open reading frames (ORFs) are indicated by specific colors according to their functional categories. The GC skew is shown as inner circles holograms in cyan and green. GC content is indicated by black circular hologram. Red; lysis-related gene. Blue; structure and packaging related genes. Yellow; nucleotide metabolism-related genes. Gray; hypothetical gene. Green; tRNA.

The whole genome of phage pSal-SNUABM-04 was sequenced and annotated (accession number; MT710307) (Figure 3). The phage genome consists of circular double-stranded DNA. The genome size was 239,626 base pairs with a GC content of 51.56%. A total of 280 putative ORFs were predicted in the genome. Most putative genes (244 of 280; 87.14%) were located on the positive strand and only 36 genes (36 of 280; 12.85%) were on the negative strand. Of these, 76 ORFs were determined to have a predicted function and 195 ORFs were classified as hypothetical proteins. The ORFs with a predicted function were classified into three categories; nucleotide metabolism-related, structure and packaging related, and lysis related genes. No genes were found to have predicted functions relating to virulence or lysogeny based on the currently available database. However, because most ORFs were predicted as hypothetical proteins, further investigation of the roles of the encoded gene products are needed.

In the phage genome, we found 10 putative tRNAs involved in phage genome transcription in the host. The presence of tRNA in the phage genome indicates phage adaptation to host bacteria. By compensating for phage codon usage, tRNA may contribute to phage infectivity and virulence.

Table S1 shows the general features of the predicted ORFs in the pSal-SNUABM-04 genome.

### 3.4. Comparative Genomics of the pSal-SNUABM-04 Genome

**Figure 4:**
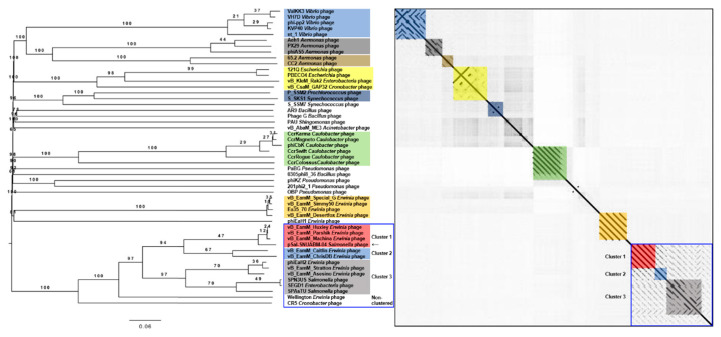
Comparative analysis of pSal-SNUABM-04 using whole-genome sequences. The phylogenetic tree was constructed using VICTOR with settings for prokaryotic viruses. The dot plot was generated with Gepard software at a word size of 10. Blue square; a large cluster composed of groups 1, 2, 3, and two non-clustered phages.

A BLAST search revealed that pSal-SNUABM-04 was highly related to the *Machinavirus* group (>96% similarity) composed of *Erwinia*-infecting jumbo-phages vB_EamM_Huxley, vB_EamM_Machina, vB_EamM_Parshik. To investigate and visualize the genomic distances between pSal-SNUABM-04 and other jumbo-phages, the phylogeny and dot plot method were performed with whole-genome and phage conserved gene sequences (major capsid protein, and terminase large subunit). The whole-genome phylogeny constructed by the VICTOR server and phylogeny-based dot plot revealed 12 clusters (Figure 4). The close-jumbo-phage cluster includes *Erwinia* phage vB_EamM_Huxley, vB_EamM_Machina, vB_EamM_Parshik, vB_EamM_ChrisDB, vB_EamM_Caitlin, Wellington, vB_EamM_Asessino, vB_EamM_Stratton, phiEaH2, *Salmonella* phage SPN3US, SPAsTU, *Enterobacteria* phage SEGD1, and *Cronobacter* phage CR5. The whole-genome phylogeny demonstrated not only the genome relationship of pSal-SNUABM-04 with *Machinavirus*, but the clue that the phage was separated recently from other cluster members. The dot plot supported the whole-genome phylogeny results, but also showed the large clustering of the group, exhibiting nucleotide similarity (blue square in Figure 4), composed of cluster 1 (vB_EamM_Huxley, vB_EamM_Machina, vB_EamM_Parshik, pSal-SNUABM-04), cluster 2 (vB_EamM_ChrisDB, vB_EamM_Caitlin), cluster 3 (vB_EamM_Asessino, vB_EamM_Stratton, phiEaH2, *Salmonella* phage SPN3US, SPAsTU, *Enterobacteria* phage SEGD1), and other non-clustered phages (Wellington and *Cronobacter* phage CR5). The phages in the big cluster also showed similar relationships in conserved gene sequence phylogeny.

**Figure 5:**
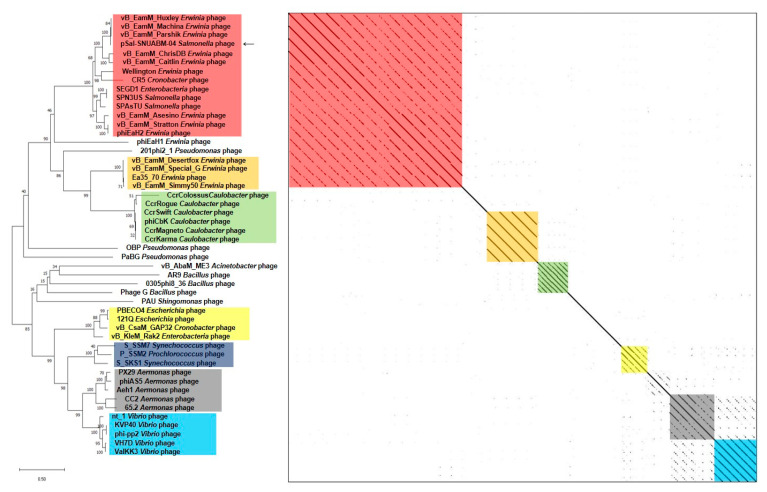
Comparative analysis of pSal-SNUABM-04 using major capsid protein sequences. The phylogenetic tree was constructed using MEGA-X by maximum likelihood with 1000 bootstrap replications. The dot plot was generated with Gepard software at a word size of 10.

**Figure 6:**
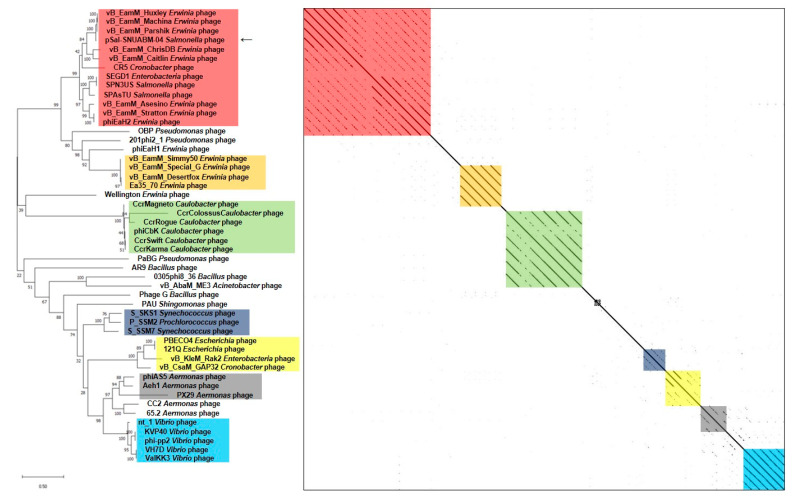
Comparative analysis of pSal-SNUABM-04 using terminase large subunit sequences. The phylogenetic tree was constructed using MEGA-X by maximum likelihood with 1000 bootstrap replications. The dot plot was generated with Gepard software at a word size of 10.

We used two gene loci, major capsid protein (Figure 5) and terminase large subunit (Figure 6), to construct the phylogenetic tree and dot plot. Although there were a few differences in the three phylogenetic trees (whole genome, major capsid protein, and terminase large subunit), the cluster containing pSal-SNUABM-04 showed high relatedness. Both single-locus phylogenies exhibited high consistency with the whole genome phylogeny, indicating that pSal-SNUABM-04 can be assigned as a new species of *Machinavirus*. 

Nucleotide homology was searched. The nucleotide and protein sequences of all ORFs were used for the BLAST search. The BLAST results showed a strong similarity between the ORFs of pSal-SNUABM-04, vB_EamM_Huxley, and other *Machinavirus* phages (Supplementary Table S1). The Ortho-average nucleotide identity (OrthoANI) values were calculated with whole-genome sequences. The calculated OrthoANI values of pSal-SNUABM-04 and *Machinavirus* jumbo-phages were remarkably high (over 94.5%), and the genomes of other close phages were <70.42% and >64.13% (Table S2). These results suggest that phage pSal-SNUABM-04 is closely associated with *Machinavirus*.

### 3.5. Specific Features of the pSal-SNUABM-04 Genome

#### 3.5.1. Nucleotide Metabolism-Related Genes

RNA polymerase (RNAP) genes were recognized by BLASTn and domain search using HHpred tool. Seven ORFs were predicted as RNAPs, i.e., ORF 30, 41, 42, 49, 223, 244, and 245. The RNAPs were assigned as two large subunits β and β’, but no recognizable σ factors were found.

The sequence similarity in phage pSal-SNUABM-04 RNAPs, and phage SPN3US RNAPs were searched in nucleotide BLAST. The three virion-associated RNAP subunit genes (vRNAP) of phage SPN3US showed high sequence similarity to three of pSal-SNUABM-04 RNAP genes, i.e., ORF 49, 244, and 245 [20]. Non-virion-associated RNAPs (nvRNAP) were not aligned. The homologous vRNAP subunits were assigned as ORF 245 to the vβN subunit, ORF 244 to vβ’N, and ORF 49 to vβ’M, whereas no C-terminal gene homologs of the β and β’ subunits were detected. However, it is reasonable to consider it to be comprised of hypothetical gene proteins. The existence of vRNAPs genes has a big meaning in phage reproduction. Because the vRNAPs injected into host bacteria start gene transcription without host transcription factors, phage progeny production becomes more independent [21,22].

The SbcCD is a complex frequently found in bacteria [20,23,24]. The complex has roles in ATP-dependent DNA repair and replication and is composed of two subunits, SbcC and SbcD. SbcC, the larger subunit, is ATPase and the SbcD subunit shows double-stranded DNA exonuclease activity [20]. SPN3US phage and other giant phages also showed homologs of the SbcCD gene [20]. In the pSal-SNUABM-04 gene, the SbcD subunit, a Mre11 nuclease domain, was found in ORF 34, and the SbcC subunit, a chromosome segregation ATPase domain, was predicted at ORF 189. The SbcCD complex is functionally grouped genes, but the homologs were placed in distance. This functional gene group splitting is a feature of phiKZ-related phage, suggesting unusual evolutional mechanisms [22].

#### 3.5.2. Structural and Packaging Related Genes

In conserved domain search, phiKZ-like internal head protein was found in the ORF 60, 61, and 98. The internal head structure, known as the inner body, is an additional substructure found in the capsids of some phiKZ-related giant phages [25]. The studies on this unusual cylindrical structure have reported that it is an essential feature of phiKZ-related phages [25,26,27,28,29,30]. However, the functions of the inner body have not been verified yet [25]. The inner body was hypothesized to have multi-function related to DNA packaging, packed genome structure, DNA ejection, or various roles in phage development [25,29].

The prohead serine protease genes, ORF 86, 123, and 249, were predicted by sequence-based searches and protein homology detection. This prohead protease plays essential role in phage capsid morphogenesis [25,30].

#### 3.5.3. Lysis-Related Genes

Of bacterial cell wall lysis genes, lytic glycosylase (ORF 200), extracellular polysaccharide (EPS) depolymerase, i.e., pectate lysate, (ORF 139) and N-acetylmuramidase (ORF 158), tail-associated lysozyme, i.e., fused to tape measure protein, (ORF 243) protein genes were identified. Phage-encoded enzymes attacking peptidoglycans were studied in many previous studies [31,32,33,34,35,36,37,38]. These enzymes have their distinct attack points which are linkage points between tetrapeptides and residues. EPS matrix secreted by bacteria makes phage hard to encounter with its targets. Therefore, these genes, especially ORF 139, verify the capacity of phage to lyse bacteria cells [36,37,38].

#### 3.5.4. Additional Function Genes

The HslV protease, heat shock-induced locus gene product V, was predicted at ORF 1 and 116 loci. The heat shock protein complex, HslVU, is an ATP-dependent protease that degrades selective proteins that are abnormal or heat-damaged [39,40]. The complex consists of two proteins, i.e., the protease subunit HslV and ATPase subunit HslU [40]. However, in the phage genome, only HslV subunit genes were encoded, whereas no HslU subunit was detected.

Bacterial ORFs encoding metal ion resistance were found in the phage genome. ORF 5 was found to be homologous to tellurite resistance protein, only *ter*B domain of the *ter* operon (*ter*Z-ABCDEF) [41]. Of the *ter* operons, *ter*B is an amphitropic peripheral membrane protein of bacterial cells [42]. A previous study suggested that *ter*B protein may contribute to the reduction of tellurite [42].

#### 3.5.5. Horizontal Gene Transfer

By metagenomic analysis with mauve system, genomes of *Machinavirus* and pSal-SNUABM-04 were compared (Figure S1). These phages showed high similarity, i.e., approximately 94% OrthoANI, although pSal-SNUABM-04 phage was isolated by using *Salmonella* host. By sequence-based homolog searching, only approximately 7% (19/270) of pSal-SNUABM-04 ORFs were hits for genes of other giant phages as the closest homologs. Most of pSal-SNUABM-04 ORFs were highly related to *Machinavirus,* but seven ORFs (3, 70, 73, 103, 169, 174, and 178) showed very low similarity. In nucleotide and protein sequence-based searching, the homologs of ORFs were not identified except for ORF 70, which was predicted as a DNA ligase. The ORFs, which showed low similarity, underwent horizontal gene transfer [42,43,44,45]. Although it is not possible to verify where these genes were transferred, it is reasonable that these transferred genes would make genomic and phenotypic differences in phage pSal-SNUABM-04 from the other *Machinavirus* members.

## 4. Conclusions

As antibiotic resistance has become a crucial issue worldwide, many studies have focused on alternatives to antibiotics [1,2]. Of the several alternatives, the bacteriophage is considered the most promising biocontrol agent owing to its many advantages such as the biosafety of in vivo phage administration and host bacteria-specific virulence [1,2]. Therefore, many studies on phages have been conducted globally [1,2,46,47,48,49,50,51]. A variety of phages have been isolated and characterized and their applications have been evaluated in many fields, including food safety, medicine, and aquacultures [46,47,48,49,50,51]. In addition, studies to overcome obstacles related to phage treatment were recently reported [52,53].

A few large phages with genomes greater than 200 kbp have been identified [3,4,5]. These large phages, so-called jumbo-phages, contain several genes responsible for phage replication and their structures [3,4,5]. These functional genes in the jumbo-phage genome provide more host independence and wider host ranges compared to normal phage [3]. Therefore, studies of jumbo-phages may reveal additional information such as phage infection strategies, virion structure, and evolution [3].

In this study, we isolated a novel *Salmonella* phage, pSal-SNUABM-04, and characterized its biological and genomic properties. This phage showed vigorous lysis efficacy against *Salmonella* bacteria cells (Figure 1D). The ability of the phage to infect a wide host range likely involves many genes encoding factors important in DNA replication and nucleotide metabolism, similar to other jumbo-phages examined in previous studies [1,4,5].

Genetic characterization revealed that pSal-SNUABM-04 is a potent new member of *Machinavirus* (Figure 4, Figure 5 and Figure 6). Most phage pSal-SNUABM-04 genes were found to be homologous to *Machinavirus* genes. Additionally, the phylogenies and dot plots constructed from the whole genome, major capsid protein, and terminase large protein support the relationship between pSal-SNUABM-04 and *Machinavirus* members. Furthermore, the comparative analysis indicated horizontal gene transfers (Figure S1).

## Figures and Tables

**Table 1:** Bacterial strains and bacterial susceptibility to pSal-SNUABM-04. −, plaque not formed; +, turbid plaque formation; ++, clear plaque formation; +++, more clear plaque formation.

Bacterial Strain	Sampled Species		pSal-SNUABM-04 Susceptibility
	Common Name	Nomenclature	
Sal-SNUABM-svn1	Savannah monitor	*Varanus exanthematicus*	+++
Sal-SNUABM-svn2	Savannah monitor	*Varanus exanthematicus*	-
Sal-SNUABM-svn3	Savannah monitor	*Varanus exanthematicus*	+
Sal-SNUABM-svn4	Savannah monitor	*Varanus exanthematicus*	+
Sal-SNUABM-lp1	Leopard gecko	*Eublepharis macularius*	+++
Sal-SNUABM-bts1	Common blue tongue skink	*Tiliqua scincoides*	-
Sal-SNUABM-mk1	Milk snake	*Lampropeltis triangulum*	+++
Sal-SNUABM-bks1	Black king snake	*Lampropeltis getula nigrita*	-
Sal-SNUABM-gg1	Tokay gecko	*gecko gekko*	-
Sal-SNUABM-bd1	Bearded dragon	*Pogona vitticeps*	+++
Sal-SNUABM-bd2	Bearded dragon	*Pogona vitticeps*	+
Sal-SNUABM-bd3	Bearded dragon	*Pogona vitticeps*	++
Sal-SNUABM-bs1	Black rat snake	*Pantherophis obsoletus*	+
Sal-SNUABM-bp1	Ball python	*Python regius*	+

## Data Availability

The data presented in this study are available in supplementary material here.

## Supplementary Materials

The following are available online at https://www.mdpi.com/1999-4915/13/1/27/s1, Figure S1: Whole genome comparison of phages in Machinavirus cluster, Table S1: Functional categories and homologies of the predicted open reading frames (ORFs) in pSal-SNUABM-04, Table S2: Calculations of average nucleotide identity (ANI) between pSal-SNUABM-04 and other jumbo-phages.
