# Supplementary material for: Isolation and Characterization of Salmonella Jumbo-Phage pSal-SNUABM-04
Source: Viruses. 2020 Dec 25;13(1):27. doi: 10.3390/v13010027 (PMC7823757; doi:10.3390/v13010027)
Supplement: Supplementary file 1 [file viruses-13-00027-s001.zip › Supplementary_files.pdf]

Supplementary Table S1. Functional categories and homologies of the predicted open reading frames (ORFs) in pSal-SNUABM-04.

Functional group	Locus tag	Encoded protein[1] [Conserved domain[2]]	Best-matched Organism	BLASTn Identity
	pSAL_SNUABM04_001	hypothetical protein [protease_HslV (cd01913); CDD_v3.18, Proteasome (PF00227.27); Pfam-A_v33.1]	[Erwinia phage vB_EamM_Parshik]	98.09%
	pSAL_SNUABM04_002	hypothetical protein	[Erwinia phage vB_EamM_Huxley]	85.71%
	pSAL_SNUABM04_003	hypothetical protein	[Erwinia phage vB_EamM_Stratton]	65.04%
	pSAL_SNUABM04_004	hypothetical protein	[Erwinia phage vB_EamM_Parshik]	86.30%
Bacterial gene	pSAL_SNUABM04_005	tellurite resistance TerB family protein [TerB (cd07176); CDD_v3.18, TerB (PF050099); Pfam-A_v33.1]	[Pantoea sp. AV62]	77.62%
	pSAL_SNUABM04_006	hypothetical protein [DUF3927 domain containing protein (PF13064.7); Pfam-A_v33.1]	[Erwinia phage vB_EamM_Huxley]	96.67%
	pSAL_SNUABM04_007	hypothetical protein [PBP2_TtGluBP (cd13567); CDD_v3.18, NMT1 like family (PF16868.6); Pfam-A_v33.1]	[Erwinia phage vB_EamM_Huxley]	99.62%
	pSAL_SNUABM04_008	hypothetical protein	[Erwinia phage vB_EamM_Huxley]	93.75%
DNA replication and nucleotide metabolism protein	pSAL_SNUABM04_009	putative chromosome partitioning protein ParB [Thermus thermophilus stage 0 sporulation protein J-like N-terminal domain, ParB family member (cd16393) containing protein; CDD_v3.18, ParB-like nuclease domain (PF02195) containing protein; Pfam-A_v33.1]	[Erwinia phage vB_EamM_Parshik]	98.15%
	pSAL_SNUABM04_010	hypothetical protein	[Erwinia phage vB_EamM_Huxley]	96.67%
	pSAL_SNUABM04_011	hypothetical protein	[Erwinia phage vB_EamM_Parshik]	96.93%
	pSAL_SNUABM04_012	hypothetical protein	[Erwinia phage vB_EamM_Huxley]	93.84%
	pSAL_SNUABM04_013	hypothetical protein	[Erwinia phage vB_EamM_Huxley]	100.00%
	pSAL_SNUABM04_014	hypothetical protein	[Erwinia phage vB_EamM_Parshik]	99.63%

	pSAL_SNUABM04_015	hypothetical protein	[Erwinia phage vB_EamM_Huxley]	96.67%
	pSAL_SNUABM04_016	hypothetical protein	[Erwinia phage Machina]	98.16%
	pSAL_SNUABM04_017	hypothetical protein	[Erwinia phage vB_EamM_Parshik]	98.98%
	pSAL_SNUABM04_018	hypothetical protein	[Erwinia phage vB_EamM_Huxley]	100.00%
	pSAL_SNUABM04_019	hypothetical protein	[Erwinia phage vB_EamM_Huxley]	97.62%
	pSAL_SNUABM04_020	hypothetical protein	[Erwinia phage vB_EamM_Huxley]	97.89%
	pSAL_SNUABM04_021	hypothetical protein	[Erwinia phage vB_EamM_Huxley]	97.22%
	pSAL_SNUABM04_022	hypothetical protein	[Erwinia phage vB_EamM_Parshik]	96.46%
	pSAL_SNUABM04_023	hypothetical protein	[Erwinia phage vB_EamM_Huxley]	100.00%
	pSAL_SNUABM04_024	hypothetical protein [POLBc_alpha; DNA polymerase type-B alpha subfamily catalytic domain (cd05532) containing protein; CDD_v3.18, DNA_pol_B; DNA polymerase family B (PF00136.22) containing protein; Pfam-A_v33.1]	[Erwinia phage vB_EamM_Parshik]	99.60%
	pSAL_SNUABM04_025	hypothetical protein	[Erwinia phage vB_EamM_Parshik]	89.91%
	pSAL_SNUABM04_026	hypothetical protein	[Erwinia phage vB_EamM_Huxley]	98.28%
	pSAL_SNUABM04_027	hypothetical protein	[Erwinia phage vB_EamM_Huxley]	95.88%
	pSAL_SNUABM04_028	hypothetical protein	[Erwinia phage vB_EamM_Huxley]	99.15%
	pSAL_SNUABM04_029	hypothetical protein	[Erwinia phage vB_EamM_Huxley]	98.01%
DNA replication and nucleotide metabolism protein	pSAL_SNUABM04_030	putative DNA-directed RNA polymerase beta subunit [largest subunit (beta') of bacterial DNA-dependent RNA polymerase N-terminal domain (cd01609); CDD_v3.18]	[Erwinia phage vB_EamM_Huxley]	100.00%
	pSAL_SNUABM04_031	hypothetical protein	[Erwinia phage vB_EamM_Huxley]	100.00%

	pSAL_SNUABM04_032	hypothetical protein	[Erwinia phage vB_EamM_Huxley]	100.00%
	pSAL_SNUABM04_033	hypothetical protein	[Erwinia phage vB_EamM_Huxley]	99.52%
DNA replication and nucleotide metabolism protein	pSAL_SNUABM04_034	putative nuclease SbcCD D subunit [MPP_Mre11_N; Mre11 nuclease, N-terminal metallophosphatase domain (cd00840) containing protein; CDD_v3.18]	[Erwinia phage vB_EamM_Huxley]	99.23%
	pSAL_SNUABM04_035	hypothetical protein	[Erwinia phage vB_EamM_Huxley]	99.29%
	pSAL_SNUABM04_036	hypothetical protein	[Erwinia phage vB_EamM_Huxley]	98.62%
	pSAL_SNUABM04_037	hypothetical protein	[Erwinia phage vB_EamM_Huxley]	99.26%
	pSAL_SNUABM04_038	hypothetical protein	[Erwinia phage vB_EamM_Huxley]	98.23%
	pSAL_SNUABM04_039	hypothetical protein	[Erwinia phage vB_EamM_Huxley]	99.78%
	pSAL_SNUABM04_040	hypothetical protein	[Erwinia phage vB_EamM_Huxley]	100.00%
DNA replication and nucleotide metabolism protein	pSAL_SNUABM04_041	putative DNA-directed RNA polymerase beta subunit [RNA polymerase beta subunit (cd00653); CDD_v3.18, RNA polymerase Rpb2_6 (PF00562.29), and RNA polymerase Rpb2_7 (PF04560.21) domain containing protein; Pfam-A_v33.1]	[Erwinia phage vB_EamM_Huxley]	99.86%
DNA replication and nucleotide metabolism protein	pSAL_SNUABM04_042	putative DNA-directed RNA polymerase beta subunit [RNAP_beta'_N (cd01609), RNAP_beta'_C (cd02655) containing protein; CDD_v3.18, RNA polymerase Rpb1, domain 3 (PF04983), domain 4 (PF05000), domain 5 (PF04998) containing protein; Pfam-A_v33.1]	[Erwinia phage vB_EamM_Huxley]	99.57%
DNA replication and nucleotide metabolism protein	pSAL_SNUABM04_043	putative ATP-dependent DNA helicase [Cas system-associated protein Cas3 type I (cd09639) containing protein; CDD_v3.18]	[Erwinia phage vB_EamM_Parshik]	99.47%
	pSAL_SNUABM04_044	hypothetical protein	[Erwinia phage vB_EamM_Huxley]	98.85%
	pSAL_SNUABM04_045	hypothetical protein	[Erwinia phage vB_EamM_Huxley]	96.53%
	pSAL_SNUABM04_046	hypothetical protein	[Erwinia phage Machina]	98.52%

	pSAL_SNUABM04_047	hypothetical protein	[Erwinia phage vB_EamM_Huxley]	100.00%
	pSAL_SNUABM04_048	hypothetical protein	[Erwinia phage vB_EamM_Huxley]	99.30%
DNA replication and nucleotide metabolism protein	pSAL_SNUABM04_049	putative DNA-directed RNA polymerase beta subunit [RNAP_largest_subunit_N (cd00399), RNAP_largest_subunit_C (cd00630) containing protein; CDD_v3.18, RNA polymerase Rpb1_domain 3 (PF04983), domain 4 (PF05000), domain 5 (PF04998); Pfam-A_v33.1]	[Erwinia phage vB_EamM_Parshik]	99.77%
	pSAL_SNUABM04_050	hypothetical protein	[Erwinia phage vB_EamM_Huxley]	95.86%
DNA replication and nucleotide metabolism protein	pSAL_SNUABM04_051	putative B family DNA polymerase [DNA_polB_II_exonuclease domain (cd05784), catalytic domain_Pol_II (cd05537) containing protein; CDD_v3.18]	[Erwinia phage vB_EamM_Huxley]	99.49%
structural and packaging protein	pSAL_SNUABM04_052	putative virion structural protein [M48 subfamily A-like (cd07345) containing protein; CDD_v3.18, Peptidase family M48 (PF01435) containing protein; Pfam-A_v33.1]	[Erwinia phage vB_EamM_Huxley]	98.45%
	pSAL_SNUABM04_053	hypothetical protein	[Erwinia phage vB_EamM_Huxley]	100.00%
	pSAL_SNUABM04_054	hypothetical protein	[Erwinia phage vB_EamM_Huxley]	98.63%
structural and packaging protein	pSAL_SNUABM04_055	putative virion structural protein	[Erwinia phage Machina]	99.49%
structural and packaging protein	pSAL_SNUABM04_056	putative virion structural protein	[Erwinia phage vB_EamM_Huxley]	100.00%
structural and packaging protein	pSAL_SNUABM04_057	putative virion structural protein	[Erwinia phage vB_EamM_Huxley]	98.55%
structural and packaging protein	pSAL_SNUABM04_058	putative virion structural protein [DUF5667 domain (PF18915.1) containing protein; Pfam-A_v33.1]	[Erwinia phage vB_EamM_Huxley]	99.04%
	pSAL_SNUABM04_059	hypothetical protein	[Erwinia phage vB_EamM_Huxley]	99.46%
	pSAL_SNUABM04_060	hypothetical protein phiKZ-like phage internal head protein (PF12699.8); Pfam-A_v33.1	[Erwinia phage vB_EamM_Huxley]	98.60%
	pSAL_SNUABM04_061	hypothetical protein phiKZ-like phage internal head protein (PF12699.8); Pfam-A_v33.1	[Erwinia phage Machina]	88.97%

	pSAL_SNUABM04_062	hypothetical protein	[Erwinia phage vB_EamM_Huxley]	93.81%
	pSAL_SNUABM04_063	hypothetical protein	[Erwinia phage vB_EamM_Huxley]	90.91%
DNA replication and nucleotide metabolism protein	pSAL_SNUABM04_064	putative dihydrofolate reductase [Dihydrofolate reductase (cd00209, PF00186.20); CDD_v3.18, Pfam-A_v33.1]	[Erwinia phage vB_EamM_Huxley]	98.33%
	pSAL_SNUABM04_065	hypothetical protein	[Erwinia phage vB_EamM_Huxley]	98.03%
structural and packaging protein	pSAL_SNUABM04_066	putative virion structural protein	[Erwinia phage Machina]	98.83%
	pSAL_SNUABM04_067	hypothetical protein	[Erwinia phage vB_EamM_Huxley]	99.44%
structural and packaging protein	pSAL_SNUABM04_068	putative virion structural protein	[Erwinia phage vB_EamM_Huxley]	99.54%
	pSAL_SNUABM04_069	hypothetical protein	[Erwinia phage vB_EamM_Huxley]	98.11%
DNA replication and nucleotide metabolism protein	pSAL_SNUABM04_070	putative DNA ligase [NAD+ dependent DNA ligase adenylation domain (cd00114) containing protein; CDD_v3.18, NAD-dependent DNA ligase adenylation domain (PF01653) containing protein; Pfam-A_v33.1]	[Salmonella phage SPAsTU]	58.06%
	pSAL_SNUABM04_071	hypothetical protein	[Erwinia phage vB_EamM_Huxley]	86.44%
	pSAL_SNUABM04_072	hypothetical protein	[Erwinia phage vB_EamM_Huxley]	93.33%
	pSAL_SNUABM04_073	hypothetical protein	[Erwinia phage vB_EamM_Caitlin]	43.90%
	pSAL_SNUABM04_074	hypothetical protein	[Erwinia phage Machina]	97.48%
	pSAL_SNUABM04_075	hypothetical protein	[Erwinia phage vB_EamM_Huxley]	98.46%
	pSAL_SNUABM04_076	hypothetical protein	[Erwinia phage vB_EamM_Huxley]	82.98%
DNA replication and nucleotide metabolism protein	pSAL_SNUABM04_077	putative helicase [DnaB-like helicase N terminal doamin (PF00772), C terminal domain (PF03796) containing protein; Pfam-A_v33.1]	[Erwinia phage vB_EamM_Huxley]	99.42%
	pSAL_SNUABM04_078	hypothetical protein	[Erwinia phage vB_EamM_Huxley]	99.44%

structural and packaging protein	pSAL_SNUABM04_079	putative major capsid protein	[Erwinia phage vB_EamM_Huxley]	99.73%
	pSAL_SNUABM04_080	hypothetical protein	[Erwinia phage vB_EamM_Parshik]	84.40%
	pSAL_SNUABM04_081	hypothetical protein [DUF1640 domain (PF07798.12) containing protein; Pfam-A_v33.1]	[Erwinia phage vB_EamM_Huxley]	98.90%
	pSAL_SNUABM04_082	hypothetical protein	[Erwinia phage vB_EamM_Huxley]	99.66%
	pSAL_SNUABM04_083	hypothetical protein	[Erwinia phage vB_EamM_Parshik]	98.85%
	pSAL_SNUABM04_084	hypothetical protein	[Erwinia phage Machina]	72.73%
	pSAL_SNUABM04_085	hypothetical protein	[Erwinia phage vB_EamM_Huxley]	98.02%
structural and packaging protein	pSAL_SNUABM04_086	putative virion structural protein [T4-like capsid assemply protein; PF07230.12]	[Erwinia phage vB_EamM_Huxley]	99.15%
structural and packaging protein	pSAL_SNUABM04_087	putative virion structural protein	[Erwinia phage vB_EamM_Huxley]	99.31%
	pSAL_SNUABM04_088	hypothetical protein	[Erwinia phage vB_EamM_Huxley]	100.00%
structural and packaging protein	pSAL_SNUABM04_089	putative virion structural protein	[Erwinia phage vB_EamM_Huxley]	98.64%
	pSAL_SNUABM04_090	hypothetical protein [RuvC; Crossover junction endodeoxyribonuclease RuvC (cd16962, PF02075.18) domain protein; CDD_v3.18, Pfam-A_v33.1]	[Erwinia phage vB_EamM_Huxley]	100.00%
	pSAL_SNUABM04_091	hypothetical protein	[Erwinia phage Machina]	98.91%
	pSAL_SNUABM04_092	hypothetical protein	[Erwinia phage vB_EamM_Huxley]	89.37%
DNA replication and nucleotide metabolism protein	pSAL_SNUABM04_093	putative acetyltransferase [Acetyltransferase (GNAT) domain (PF13673.8) containing protein; Pfam-A_v33.1]	[Erwinia phage vB_EamM_Huxley]	97.47%
	pSAL_SNUABM04_094	hypothetical protein	[Erwinia phage vB_EamM_Huxley]	96.27%
	pSAL_SNUABM04_095	hypothetical protein	[Erwinia phage vB_EamM_Huxley]	99.23%
	pSAL_SNUABM04_096	hypothetical protein [YpsA SLOG family protein (PF06908.12); Pfam-A_v33.1]	[Erwinia phage vB_EamM_Parshik]	98.50%

	pSAL_SNUABM04_097	hypothetical protein	[Erwinia phage vB_EamM_Huxley]	75.28%
	pSAL_SNUABM04_098	hypothetical protein<br>phiKZ-like phage internal head protein (PF12699.8); Pfam-A_v33.1]	[Erwinia phage vB_EamM_Huxley]	99.74%
DNA replication and nucleotide metabolism protein	pSAL_SNUABM04_099	putative GNAT family acetyltransferase<br>[Acetyltransferase (GNAT) domain (PF13527) protein; Pfam-A_v33.1]	[Erwinia phage vB_EamM_Huxley]	99.37%
	pSAL_SNUABM04_100	hypothetical protein	[Erwinia phage Machina]	99.00%
	pSAL_SNUABM04_101	hypothetical protein	[Erwinia phage vB_EamM_Huxley]	99.18%
	pSAL_SNUABM04_102	hypothetical protein	[Erwinia phage Machina]	99.50%
	pSAL_SNUABM04_103	hypothetical protein	[Salmonella enterica subsp. enterica serovar Adabraka]	65.08%
DNA replication and nucleotide metabolism protein	pSAL_SNUABM04_104	putative thymidylate kinase<br>[Thymidine monophosphate kinase (cd01672) protein; CDD_v3.18, Thymidylate kinase (PF02223) protein; Pfam-A_v33.1]	[Erwinia phage Machina]	99.53%
DNA replication and nucleotide metabolism protein	pSAL_SNUABM04_105	putative transcriptional regulator<br>[Antirepressor AbbA (PF14156) containing protein; Pfam-A_v33.1]	[Erwinia phage Machina]	98.28%
	pSAL_SNUABM04_106	hypothetical protein	[Erwinia phage vB_EamM_Huxley]	97.40%
DNA replication and nucleotide metabolism protein	pSAL_SNUABM04_107	putative GCN5-related N-acetyltransferase<br>[Acetyltransferase (GNAT) domain (PF13420) containing protein; Pfam-A_v33.1]	[Erwinia phage vB_EamM_Huxley]	97.91%
DNA replication and nucleotide metabolism protein	pSAL_SNUABM04_108	putative DNA adenine methylase<br>[D12 class N6 adenine-specific DNA methyltransferase (PF02086.16) protein; Pfam-A_v33.1]	[Erwinia phage vB_EamM_Huxley]	98.90%
	pSAL_SNUABM04_109	hypothetical protein	[Erwinia phage vB_EamM_Huxley]	98.86%
	pSAL_SNUABM04_110	hypothetical protein	[Erwinia phage vB_EamM_Huxley]	98.42%
	pSAL_SNUABM04_111	hypothetical protein	[Erwinia phage vB_EamM_Huxley]	100.00%
	pSAL_SNUABM04_112	hypothetical protein	[Erwinia phage vB_EamM_Huxley]	99.40%

	pSAL_SNUABM04_113	hypothetical protein	[Erwinia phage vB_EamM_Huxley]	95.50%
	pSAL_SNUABM04_114	hypothetical protein	[Salmonella enterica subsp. enterica serovar Heidelberg]	76.15%
	pSAL_SNUABM04_115	hypothetical protein	[Erwinia phage vB_EamM_Huxley]	94.33%
	pSAL_SNUABM04_116	hypothetical protein [protease_HslV (cd01913) protein; CDD_v3.18, proteasome (PF00227) protein; Pfam-A_v33.1]	[Erwinia phage vB_EamM_Huxley]	97.71%
	pSAL_SNUABM04_117	hypothetical protein	[Erwinia phage vB_EamM_Huxley]	95.05%
	pSAL_SNUABM04_118	hypothetical protein	[Erwinia phage vB_EamM_Parshik]	97.54%
	pSAL_SNUABM04_119	hypothetical protein	[Erwinia phage Machina]	98.18%
	pSAL_SNUABM04_120	hypothetical protein	[Erwinia phage vB_EamM_Huxley]	96.81%
DNA replication and nucleotide metabolism related protein	pSAL_SNUABM04_121	putative dnaA protein [DNA replication licensing factor Mcm4 (cd17755) protein; CDD_v3.18, bacterial dnaA (PF00308) protein; Pfam-A_v33.1]	[Erwinia phage vB_EamM_Huxley]	97.22%
	pSAL_SNUABM04_122	hypothetical protein	Erwinia phage vB_EamM_Parshik	92.31%
	pSAL_SNUABM04_123	hypothetical protein	[Erwinia phage vB_EamM_Huxley]	91.72%
	pSAL_SNUABM04_124	hypothetical protein	[Erwinia phage Machina]	71.21%
	pSAL_SNUABM04_125	hypothetical protein	[Erwinia phage vB_EamM_Huxley]	96.55%
	pSAL_SNUABM04_126	hypothetical protein [ExsB domain (cd01995) containing protein; CDD_v3.18, QueC domain (PF06508) containing protein; Pfam-A_v33.1]	[Erwinia phage vB_EamM_Parshik]	97.94%
	pSAL_SNUABM04_127	hypothetical protein [DUF488 domain (PF04343.14) containing protein; Pfam-A_v33.1]	[Erwinia phage vB_EamM_Parshik]	98.78%
	pSAL_SNUABM04_128	hypothetical protein	[Erwinia phage vB_EamM_Huxley]	95.38%
	pSAL_SNUABM04_129	hypothetical protein	[Erwinia phage vB_EamM_Huxley]	71.67%

	pSAL_SNUABM04_130	hypothetical protein	[Erwinia phage vB_EamM_Huxley]	98.84%
	pSAL_SNUABM04_131	hypothetical protein	[Erwinia phage vB_EamM_Huxley]	94.51%
	pSAL_SNUABM04_132	hypothetical protein	[Erwinia phage vB_EamM_Huxley]	98.15%
	pSAL_SNUABM04_133	hypothetical protein	[Erwinia phage vB_EamM_Huxley]	88.49%
	pSAL_SNUABM04_134	hypothetical protein	[Erwinia phage vB_EamM_Huxley]	95.68%
	pSAL_SNUABM04_135	hypothetical protein	[Erwinia phage vB_EamM_Huxley]	75.17%
	pSAL_SNUABM04_136	hypothetical protein	[Erwinia phage Machina]	72.02%
	pSAL_SNUABM04_137	hypothetical protein	[Erwinia phage Machina]	75.18%
	pSAL_SNUABM04_138	hypothetical protein	[Erwinia phage vB_EamM_Huxley]	91.78%
lysis related protein	pSAL_SNUABM04_139	putative EPS depolymerase [Pectate lysate superfamily domain (PF12708.8) cotaining protein; Pfam-A_v33.1]	[Erwinia phage vB_EamM_Parshik]	96.66%
structural and packaging protein	pSAL_SNUABM04_140	putative virion structural protein	[Erwinia phage vB_EamM_Parshik]	73.11%
structural and packaging protein	pSAL_SNUABM04_141	putative virion structural protein	[Erwinia phage vB_EamM_Stratton]	74.17%
structural and packaging protein	pSAL_SNUABM04_142	putative virion structural protein	[Erwinia phage vB_EamM_Asesino]	72.89%
structural and packaging protein	pSAL_SNUABM04_143	putative virion structural protein	[Erwinia phage vB_EamM_Asesino]	78.95%
	pSAL_SNUABM04_144	hypothetical protein	[Erwinia phage vB_EamM_Asesino]	78.49%
structural and packaging protein	pSAL_SNUABM04_145	putative virion structural protein	[Erwinia phage vB_EamM_Asesino]	71.53%
structural and packaging protein	pSAL_SNUABM04_146	putative virion structural protein	[Erwinia phage vB_EamM_Asesino]	72.24%
structural and packaging protein	pSAL_SNUABM04_147	putative virion structural protein	[Erwinia phage vB_EamM_Asesino]	43.42%
structural and packaging protein	pSAL_SNUABM04_148	putative virion structural protein	[Erwinia phage vB_EamM_Asesino]	51.92%
structural and packaging protein	pSAL_SNUABM04_149	putative virion structural protein	[Erwinia phage vB_EamM_Asesino]	46.96%

structural and packaging protein	pSAL_SNUABM04_150	putative virion structural protein	[Erwinia phage vB_EamM_ChrisDB	48.09%
structural and packaging protein	pSAL_SNUABM04_151	putative virion structural protein	[Erwinia phage vB_EamM_Asesino]	53.87%
structural and packaging protein	pSAL_SNUABM04_152	putative virion structural protein [Fibronectin-III type domain (PF16794.6) containing protein; Pfam-A_v33.1]	[Cronobacter phage CR5]	47.12%
	pSAL_SNUABM04_153	hypothetical protein [LPAM_1 domain (PF08139.13) containing protein; Pfam-A_v33.1]	[Erwinia phage vB_EamM_Huxley]	92.47%
	pSAL_SNUABM04_154	hypothetical protein	[Erwinia phage vB_EamM_Huxley]	85.96%
	pSAL_SNUABM04_155	hypothetical protein	[Erwinia phage vB_EamM_Huxley]	91.57%
	pSAL_SNUABM04_156	hypothetical protein [DUF1353 domain (PF07087.12) containing protein]	[Erwinia phage vB_EamM_Huxley]	96.61%
	pSAL_SNUABM04_157	hypothetical protein	[Erwinia phage vB_EamM_Huxley]	97.37%
lysis related protein	pSAL_SNUABM04_158	putative peptidoglycan domain protein [N-acetylmuramidase_GH108 domain (cd13926) containing protein; CDD_v3.18, Glyco_hydro_108 domain (PF05838.13), PG_binding_3 domain (PF09374.11) containing protein; Pfam-A_v33.1]	[Erwinia phage vB_EamM_Huxley]	99.50%
	pSAL_SNUABM04_159	hypothetical protein [DUF4376 domain (PF14301.7) containing protein; Pfam-A_v33.1]	[Erwinia phage vB_EamM_Parshik]	98.62%
	pSAL_SNUABM04_160	hypothetical protein	[Erwinia phage vB_EamM_Huxley]	98.92%
DNA replication and nucleotide metabolism protein	pSAL_SNUABM04_161	putative radical SAM superfamily protein	[Erwinia phage vB_EamM_Huxley]	99.33%
DNA replication and nucleotide metabolism protein	pSAL_SNUABM04_162	putative radical SAM superfamily protein	[Erwinia phage vB_EamM_Huxley]	99.62%
	pSAL_SNUABM04_163	hypothetical protein	[Erwinia phage vB_EamM_Parshik]	96.58%
	pSAL_SNUABM04_164	hypothetical protein	[Erwinia phage vB_EamM_Huxley]	100.00%
	pSAL_SNUABM04_165	hypothetical protein	[Erwinia phage vB_EamM_Parshik]	96.40%
	pSAL_SNUABM04_166	hypothetical protein	[Erwinia phage vB_EamM_Parshik]	96.88%

	pSAL_SNUABM04_167	hypothetical protein	[Erwinia phage vB_EamM_Parshik]	98.25%
	pSAL_SNUABM04_168	hypothetical protein [Uncharacterised protein family domain (UPF0242) containing protein; Pfam-A_v33.1]	[Erwinia phage vB_EamM_Huxley]	74.27%
structural and packaging protein	pSAL_SNUABM04_169	hypothetical protein	[Erwinia phage vB_EamM_Parshik]	97.28%
structural and packaging protein	pSAL_SNUABM04_170	putative virion structural protein	[Erwinia phage vB_EamM_Huxley]	97.03%
structural and packaging protein	pSAL_SNUABM04_171	putative virion structural protein	[Erwinia phage Machina]	98.81%
structural and packaging protein	pSAL_SNUABM04_172	putative virion structural protein	[Erwinia phage vB_EamM_Huxley]	100.00%
	pSAL_SNUABM04_173	hypothetical protein [DUF1471 domain (PF07338.14) containing protein; Pfam-A_v33.1]	[Chloroflexi bacterium]	35.29%
	pSAL_SNUABM04_174	hypothetical protein	[Erwinia phage Machina]	98.05%
structural and packaging protein	pSAL_SNUABM04_175	putative virion structural protein [Bacteriophage Mu Gp45 spike protein (PF06890) domain containing protein; Pfam-A_v33.1]	[Erwinia phage vB_EamM_Huxley]	96.17%
	pSAL_SNUABM04_176	hypothetical protein	[Erwinia phage Machina]	86.83%
	pSAL_SNUABM04_177	hypothetical protein	[Erwinia phage Machina]	31.25%
	pSAL_SNUABM04_178	hypothetical protein	[Erwinia phage vB_EamM_Huxley]	61.19%
	pSAL_SNUABM04_179	hypothetical protein	[Erwinia phage vB_EamM_Parshik]	92.66%
	pSAL_SNUABM04_180	hypothetical protein	[Erwinia phage vB_EamM_Huxley]	97.86%
	pSAL_SNUABM04_181	hypothetical protein [ART-PolyVal domain (PF18760) containing protein; Pfam-A_v33.1]	[Erwinia phage Machina]	96.32%
	pSAL_SNUABM04_182	hypothetical protein	[Erwinia phage vB_EamM_Huxley]	54.30%
	pSAL_SNUABM04_183	hypothetical protein	[Erwinia phage vB_EamM_Huxley]	94.49%
	pSAL_SNUABM04_184	hypothetical protein	[Erwinia phage vB_EamM_Huxley]	99.25%

	pSAL_SNUABM04_185	hypothetical protein [PAGK domain (PF15284) containing protein; Pfam-A_v33.1]	[Erwinia phage vB_EamM_Huxley]	100.00%
	pSAL_SNUABM04_186	hypothetical protein	[Erwinia phage vB_EamM_Huxley]	93.89%
	pSAL_SNUABM04_187	hypothetical protein	[Erwinia phage vB_EamM_Huxley]	94.24%
	pSAL_SNUABM04_188	hypothetical protein	[Erwinia phage Machina]	98.45%
DNA replication and nucleotide metabolism protein	pSAL_SNUABM04_189	putative SMC domain-containing protein [putative Smc; Chromosome segregation ATPase domain (COG1196) protein; COG_KOG_v1.0]	[Erwinia phage Machina]	94.74%
	pSAL_SNUABM04_190	hypothetical protein	[Erwinia phage Machina]	100.00%
	pSAL_SNUABM04_191	hypothetical protein	[Erwinia phage vB_EamM_Huxley]	73.85%
	pSAL_SNUABM04_192	hypothetical protein	[Erwinia phage vB_EamM_Huxley]	95.18%
	pSAL_SNUABM04_193	hypothetical protein	[Erwinia phage vB_EamM_Huxley]	95.03%
DNA replication and nucleotide metabolism protein	pSAL_SNUABM04_194	putative acethyltransferase [Acetyltransferase (PF13420) domain protein; Pfam-A_v33.1]	[Erwinia phage vB_EamM_Huxley]	94.55%
DNA replication and nucleotide metabolism protein	pSAL_SNUABM04_195	putative GTPase with similarity to GTPase center in EF-Tu [EF-Tu_GTP-binding domain (cd01884) conteining protein; CDD_v3.18, Pfam-A_v33.1]	[Erwinia phage vB_EamM_Huxley]	98.82%
	pSAL_SNUABM04_196	hypothetical protein	[Erwinia phage vB_EamM_Huxley]	96.15%
	pSAL_SNUABM04_197	hypothetical protein	[Erwinia phage vB_EamM_Huxley]	97.73%
	pSAL_SNUABM04_198	hypothetical protein	[Erwinia phage vB_EamM_Huxley]	98.76%
structural and packaging protein	pSAL_SNUABM04_199	putative Appr-1-p processing protein [macrodomain superfamily domain (cd02749) protein; CDD_v3.18]	[Erwinia phage vB_EamM_Huxley]	100.00%
lysis related protein	pSAL_SNUABM04_200	putative endolysin [LT_MltC_MltE domain (cd16893) containing protein; CDD_v3.18]	[Erwinia phage vB_EamM_Huxley]	98.24%
	pSAL_SNUABM04_201	hypothetical protein	[Erwinia phage vB_EamM_Huxley]	96.30%

	pSAL_SNUABM04_202	hypothetical protein	[Erwinia phage vB_EamM_Huxley]	86.10%
	pSAL_SNUABM04_203	hypothetical protein	[Erwinia phage vB_EamM_Huxley]	94.88%
	pSAL_SNUABM04_204	hypothetical protein	[Erwinia phage vB_EamM_Huxley]	98.05%
	pSAL_SNUABM04_205	hypothetical protein	[Erwinia phage vB_EamM_Huxley]	100.00%
structural and packaging protein	pSAL_SNUABM04_206	putative virion structural protein	[Erwinia phage vB_EamM_Parshik]	99.38%
	pSAL_SNUABM04_207	hypothetical protein	[Erwinia phage vB_EamM_Huxley]	99.35%
	pSAL_SNUABM04_208	hypothetical protein	[Erwinia phage vB_EamM_Huxley]	99.60%
DNA replication and nucleotide metabolism protein	pSAL_SNUABM04_209	putative endodeoxyribonuclease [DUF5051 ; 3' exoribonuclease, RNase T-like domain (PF16473.6) containing protein; Pfam-A_v33.1]	[Erwinia phage vB_EamM_Huxley]	99.41%
DNA replication and nucleotide metabolism protein	pSAL_SNUABM04_210	putative ribonuclease H [RNase_HI_prokaryote_like domain (cd09278) containing protein; CDD_v3.18]	[Erwinia phage Machina]	99.60%
	pSAL_SNUABM04_211	hypothetical protein	[Erwinia phage vB_EamM_Huxley]	97.95%
	pSAL_SNUABM04_212	hypothetical protein	[Erwinia phage vB_EamM_Huxley]	99.18%
	pSAL_SNUABM04_213	hypothetical protein	[Erwinia phage vB_EamM_Huxley]	97.55%
	pSAL_SNUABM04_214	hypothetical protein	[Erwinia phage vB_EamM_Huxley]	97.67%
	pSAL_SNUABM04_215	hypothetical protein	[Erwinia phage vB_EamM_Huxley]	97.77%
	pSAL_SNUABM04_216	hypothetical protein	[Erwinia phage vB_EamM_Huxley]	81.59%
	pSAL_SNUABM04_217	hypothetical protein	[Erwinia phage vB_EamM_Huxley]	91.82%
	pSAL_SNUABM04_218	hypothetical protein	[Erwinia phage vB_EamM_Huxley]	99.60%
	pSAL_SNUABM04_219	hypothetical protein	[Erwinia phage vB_EamM_Huxley]	100.00%
	pSAL_SNUABM04_220	hypothetical protein	[Erwinia phage vB_EamM_Huxley]	97.19%

DNA replication and nucleotide metabolism protein	pSAL_SNUABM04_221	putative RecA recombinase [RecA domain (cd00983, PF00154.22) containing protein; CDD_v3.18, Pfam-A_v33.1]	[Erwinia phage vB_EamM_Huxley]	97.85%
	pSAL_SNUABM04_222	hypothetical protein	[Erwinia phage vB_EamM_Huxley]	100.00%
DNA replication and nucleotide metabolism protein	pSAL_SNUABM04_223	putative RNA polymerase beta subunit [RNA_pol_B_RPB2 domain (cd00653) containing protein; CDD_v3.18, RNA_pol_Rpb2 domain 6 (PF00562.29), domain 7 (PF04560.21) containing protein; Pfam-A_v33.1]	[Erwinia phage vB_EamM_Huxley]	99.17%
structural and packaging protein	pSAL_SNUABM04_224	putative virion structural protein	[Erwinia phage vB_EamM_Huxley]	97.40%
	pSAL_SNUABM04_225	hypothetical protein	[Erwinia phage vB_EamM_Huxley]	99.44%
	pSAL_SNUABM04_226	hypothetical protein	[Erwinia phage vB_EamM_Huxley]	100.00%
	pSAL_SNUABM04_227	hypothetical protein	[Erwinia phage vB_EamM_Huxley]	99.32%
	pSAL_SNUABM04_228	hypothetical protein	[Erwinia phage vB_EamM_Huxley]	99.71%
	pSAL_SNUABM04_229	hypothetical protein	[Erwinia phage vB_EamM_Huxley]	100.00%
	pSAL_SNUABM04_230	hypothetical protein [Phage_30_3 domain (PF08010.12) containing protein; Pfam-A_v33.1]	[Erwinia phage Machina]	100.00%
	pSAL_SNUABM04_231	hypothetical protein	[Erwinia phage vB_EamM_Huxley]	96.26%
	pSAL_SNUABM04_232	hypothetical protein [NTP-PPase_SsMazG domain (cd11535) containing protein; CDD_v3.18]	[Erwinia phage Machina]	88.39%
DNA replication and nucleotide metabolism protein	pSAL_SNUABM04_233	putative thymidylate synthase [Thymidylat_synt domain (PF00303.20) protein; Pfam-A_v33.1, TS_Pyrimidine_HMase domain (cd00351) protein; CDD_v3.18,]	[Erwinia phage vB_EamM_Parshik]	90.96%
	pSAL_SNUABM04_234	hypothetical protein	[Erwinia phage vB_EamM_Huxley]	98.26%
	pSAL_SNUABM04_235	hypothetical protein	[Erwinia phage vB_EamM_Huxley]	94.00%
	pSAL_SNUABM04_236	hypothetical protein	[Erwinia phage vB_EamM_Huxley]	96.30%

	pSAL_SNUABM04_237	hypothetical protein	[Erwinia phage Machina]	98.92%
DNA replication and nucleotide metabolism protein	pSAL_SNUABM04_238	putative mazG nucleotide pyrophosphohydrolase [Nucleoside Triphosphate Pyrophosphohydrolase MazG-like domain (cd11543) containing protein; CDD_v3.18, MazG nucleotide pyrophosphohydrolase domain (PF03819) containing protein; Pfam-A_v33.1]	[Erwinia phage Machina]	92.61%
	pSAL_SNUABM04_239	hypothetical protein	[Erwinia phage vB_EamM_Huxley]	97.59%
	pSAL_SNUABM04_240	hypothetical protein	[Erwinia phage vB_EamM_Huxley]	97.26%
	pSAL_SNUABM04_241	hypothetical protein	[Erwinia phage vB_EamM_Huxley]	99.45%
	pSAL_SNUABM04_242	hypothetical protein	[Erwinia phage vB_EamM_Parshik]	99.07%
structural and packaging protein	pSAL_SNUABM04_243	Tail-associated lysozyme [Transglycosylase SLT domain (PF01464); Pfam-A_v33.1]	[Erwinia phage vB_EamM_Huxley]	99.81%
DNA replication and nucleotide metabolism protein	pSAL_SNUABM04_244	putative DNA-directed RNA polymerase beta subunit [RNAP_beta'_N domain (cd01609) protein; CDD_v3.18]	[Erwinia phage vB_EamM_Parshik]	99.50%
DNA replication and nucleotide metabolism protein	pSAL_SNUABM04_245	putative DNA-directed RNA polymerase beta subunit [RNA_pol_B_RPB2 domain (cd00653) containing protein; CDD_v3.18]	[Erwinia phage vB_EamM_Huxley]	100.00%
	pSAL_SNUABM04_246	hypothetical protein	[Erwinia phage vB_EamM_Huxley]	99.18%
	pSAL_SNUABM04_247	hypothetical protein	[Erwinia phage vB_EamM_Huxley]	99.58%
	pSAL_SNUABM04_248	hypothetical protein	[Erwinia phage vB_EamM_Huxley]	100.00%
structural and packaging protein	pSAL_SNUABM04_249	putative virion structural protein [Prohead core protein serine protease domain (PF03420.14) containing protein; Pfam-A_v33.1]	[Erwinia phage vB_EamM_Parshik]	100.00%
	pSAL_SNUABM04_250	hypothetical protein	[Erwinia phage vB_EamM_Huxley]	100.00%
	pSAL_SNUABM04_251	hypothetical protein	[Erwinia phage vB_EamM_Parshik]	99.46%
	pSAL_SNUABM04_252	hypothetical protein [NT5C domain (PF069441.13) containing protein; Pfam-A_v33.1]	[Erwinia phage vB_EamM_Huxley]	82.54%

	pSAL_SNUABM04_253	hypothetical protein	[Erwinia phage vB_EamM_Huxley]	98.67%
	pSAL_SNUABM04_254	hypothetical protein [DUF4202 domain (PF13875.7) contanining protein; Pfam-A_v33.1]	[Erwinia phage vB_EamM_Huxley]	100.00%
	pSAL_SNUABM04_255	hypothetical protein	[Erwinia phage vB_EamM_Huxley]	94.74%
	pSAL_SNUABM04_256	hypothetical protein	[Erwinia phage vB_EamM_Huxley]	96.63%
	pSAL_SNUABM04_257	hypothetical protein	[Erwinia phage vB_EamM_Huxley]	100.00%
structural and packaging protein	pSAL_SNUABM04_258	putative virion structural protein	[Erwinia phage vB_EamM_Parshik]	99.26%
structural and packaging protein	pSAL_SNUABM04_259	putative tail sheath protein	[Erwinia phage vB_EamM_Huxley]	99.67%
	pSAL_SNUABM04_260	hypothetical protein	[Erwinia phage vB_EamM_Parshik]	98.10%
structural and packaging protein	pSAL_SNUABM04_261	putative virion structural protein	[Erwinia phage vB_EamM_Huxley]	99.63%
structural and packaging protein	pSAL_SNUABM04_262	putative structural protein	[Erwinia phage vB_EamM_Parshik]	99.86%
structural and packaging protein	pSAL_SNUABM04_263	putative terminase large subunit [Terminase_1 domain (PF03354.16) contanining protein; Pfam-A_v33.1]	[Erwinia phage vB_EamM_Huxley]	100.00%
	pSAL_SNUABM04_264	hypothetical protein	[Erwinia phage vB_EamM_Huxley]	100.00%
	pSAL_SNUABM04_265	hypothetical protein	[Erwinia phage vB_EamM_Huxley]	98.94%
	pSAL_SNUABM04_266	hypothetical protein	[Erwinia phage vB_EamM_Huxley]	100.00%
	pSAL_SNUABM04_267	hypothetical protein	[Erwinia phage vB_EamM_Huxley]	92.75%
	pSAL_SNUABM04_268	hypothetical protein	[Erwinia phage vB_EamM_Parshik]	98.28%
	pSAL_SNUABM04_269	hypothetical protein	[Erwinia phage vB_EamM_Parshik]	98.41%
	pSAL_SNUABM04_270	hypothetical protein	[Erwinia phage vB_EamM_Huxley]	100.00%
tRNA	pSAL_SNUABM04_271	tRNA-Trp		
tRNA	pSAL_SNUABM04_272	tRNA-Cys		
tRNA	pSAL_SNUABM04_273	tRNA-Tyr		

tRNA	pSAL_SNUABM04_274	tRNA-Gln
tRNA	pSAL_SNUABM04_275	tRNA-Asp
tRNA	pSAL_SNUABM04_276	tRNA-Ile
tRNA	pSAL_SNUABM04_277	tRNA-Glu
tRNA	pSAL_SNUABM04_278	tRNA-Ser
tRNA	pSAL_SNUABM04_279	tRNA-Ile2
tRNA	pSAL_SNUABM04_280	tRNA-Asn

[1] Determined by BLASTn searching.

[2] Determined by HHpred searching.

Supplementary Table S2. Calculations of average nucleotide identity (ANI) between pSal-SNUABM-04 and other neighboring jumbo-phages.

**phages**	**OrthoANI**
vB_EamM_Machina *Erwinia* phage	94.55
vB_EamM_Parshik *Erwinia* phage	94.5
vB_EamM_Huxley *Erwinia* phage	94.63
vB_EamM_Caitlin *Erwinia* phage	70.42
vB_EamM_ChrisDB *Erwinia* phage	69.76
phiEaH2 *Erwinia* phage	67.72
vB_EamM_Stratton *Erwinia* phage	68.04
vB_EamM_Asessino *Erwinia* phage	67.28
SPN3US *Salmonella* phage	66.87
SEGD1 *Salmonella* phage	66.85
SPAsTU *Salmonella* phage	67.52
Wellington *Erwinia* phage	65.56
CR5 *Cronobacter* phage	64.13

[1] Tables may have a footer.

Supplementary Figure S1. Whole genome comparison of phages in *Machinavirus* cluster

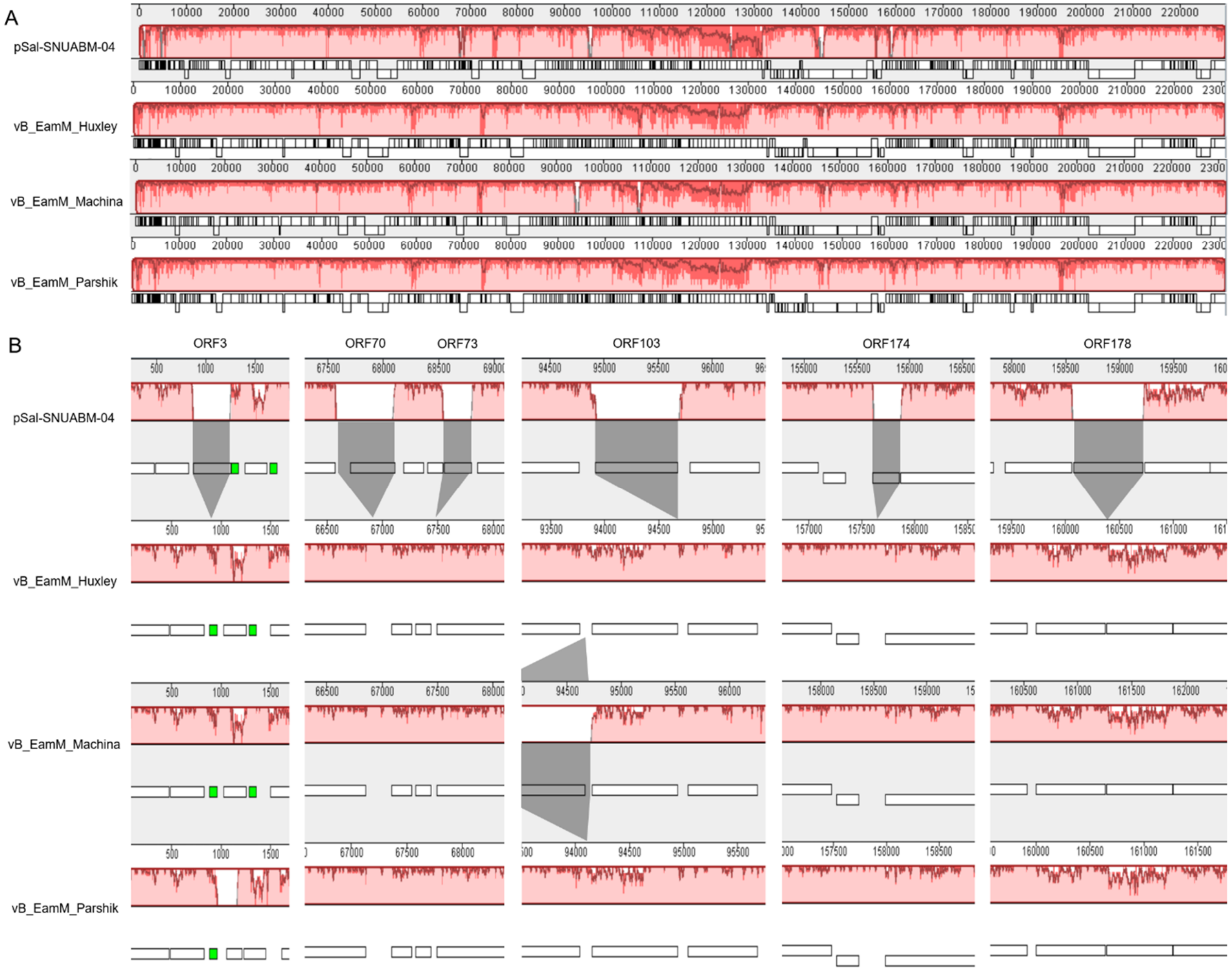

(A) Whole genome comparison of pSal-SNUABM-04, and *Machinavirus* phages. (B) Magnifications comparing the similarity of ORFs between pSal-SNUABM-04, and *Machinavirus* phages. The ORFs showed very low similarity to *Machinavirus* phages.
