# Supplementary figures and images for: Isolation and Characterization of Salmonella Jumbo-Phage pSal-SNUABM-04
Source: Viruses. 2020 Dec 25;13(1):27. doi: 10.3390/v13010027 (PMC7823757; doi:10.3390/v13010027)

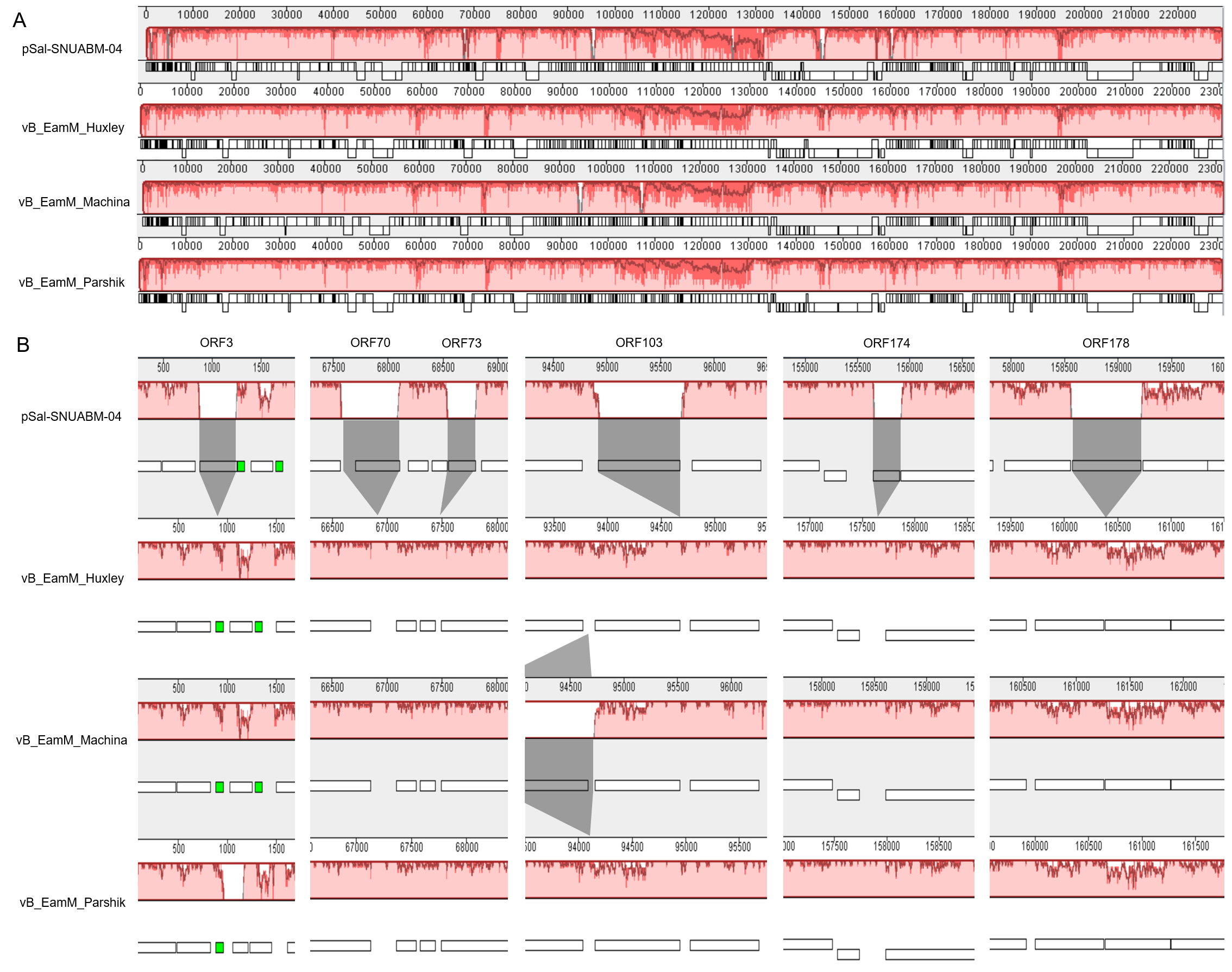

Supplement: Supplementary file 1 [file viruses-13-00027-s001.zip › Figure S1.jpg]
